# Pseudoaneurysm of the Dorsalis Pedis Artery Diagnosed on Point-of-Care Ultrasound

**DOI:** 10.24908/pocus.v6i1.14752

**Published:** 2021-04-22

**Authors:** Nathan A Friedman, Caleb P Canders, Alan T Chiem

**Affiliations:** 1 Department of Emergency Medicine, Ronald Regan UCLA Medical Center LA, CA; 2 Department of Emergency Medicine, UCLA Medical Center Olive View Sylmar, CA

**Keywords:** dorsalis pedis, pseudoaneurysm, point-of-care ultrasound, foot mass

## Abstract

A 46-year-old man presented with a painless mass on his dorsal right foot one week after striking it on a door. A traumatic hematoma was suspected, and needle aspiration of the mass is considered. However, point-of-care ultrasound performed by the emergency physician identified a pseudoaneurysm of the dorsalis pedis artery, a rare condition that can occur after minor trauma or iatrogenic intervention. This report demonstrates how point-of-care ultrasound can be used to identify a pseudoaneurysm of the lower extremity, thereby expediting emergency department workup and preventing potentially dangerous diagnostic procedures.

## Introduction

Vascular aneurysms and pseudoaneurysms of the arteries of the foot are uncommon. Pseudoaneurysms form when a hematoma accumulates between the outer layers of the artery, the tunica media and tunica adventitia, usually following a trauma. Angiography, although traditionally the gold standard for diagnosis of lower extremity pseudoaneurysms, has largely been replaced by computed tomography angiography (CTA) or duplex sonography in the emergency department (ED). Our case demonstrates how emergency physicians can apply point-of-care ultrasound to diagnose a pseudoaneurysm of the dorsalis pedis artery, thereby preventing iatrogenic injury (e.g. diagnostic aspiration or incision) and expediting appropriate follow-up.

## Case Report

**Figure 1:**
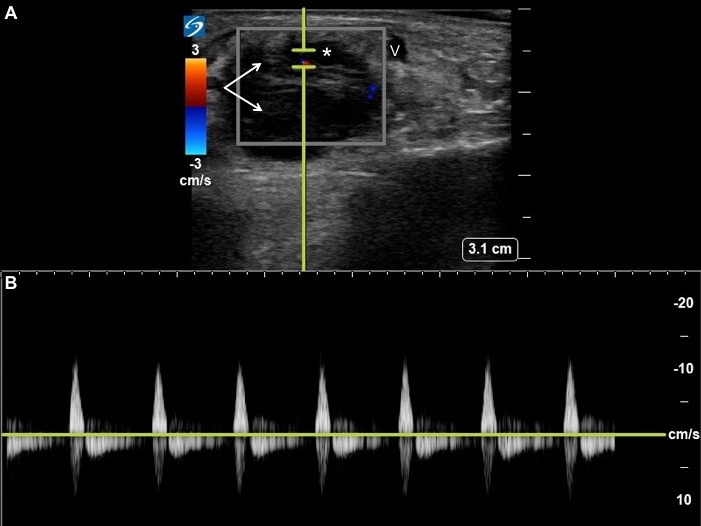
(A) Point-of-care ultrasound demonstrates a hypoechoic cystic mass (arrows) adjacent to the dorsalis pedis artery (asterisk). (B) A Doppler gate placed over the neck of the mass reveals arterial waveforms, consistent with a pseudoaneurysm of the dorsalis pedis artery.

A 46-year-old man presented to the ED with a soft tissue mass on his dorsal right foot after striking it on a door one week prior. He denied fevers, difficulty bearing weight, or associated pain or numbness in the area. On examination, he had a soft, non-tender, mobile, 3 cm x 3 cm mass on the dorsal right foot without overlying skin changes. Pulsations were felt over the dorsalis pedis artery but not the mass. The patient’s toes were well-perfused, and he had no sensory or motor deficits. Traumatic hematoma was suspected, and diagnostic aspiration was considered. However, point-of-care ultrasound performed by the emergency physician revealed a hypoechoic cystic mass, just lateral to the dorsalis pedis artery, with arterial waveforms. [Image 1A-B] The addition of color Doppler ultrasound revealed pulsatile flow within the mass, consistent with a pseudoaneurysm of the dorsalis pedis artery (Figure 1, online Video S1. The patient provided written consent for publication of the case.

## Discussion

Pseudoaneurysms of the dorsalis pedis artery comprise less than 0.5% of all lower extremity aneurysms or pseudoaneurysms 1. Foot pseudoaneurysms can occur days to years after a blunt or penetrating trauma, which may be minor 2. Iatrogenic pseudoaneurysms of the dorsalis pedis artery have also been reported after ankle arthroscopy, Lisfranc operations, and arterial line placement 3, 4, 5. Pseudoaneurysms are classically painless and pulsatile. However, compression of adjacent structures can cause pain, and thrombus formation within the pseudoaneurysm may prevent pulsatility, often making physical examination unreliable.

Angiography, despite being the gold standard imaging for patients with suspected lower extremity pseudoaneurysms, is often unavailable in the ED. CTA, although commonly performed in the workup of suspected pseudoaneurysms, is potentially nephrotoxic, limited by its resolution at the distal arteries, and has reconstruction-based artifacts 6. Sonography with color Doppler, on the other hand, is radiation-free, readily available, and able to distinguish pseudoaneurysms from hematomas, abscesses, neoplasms, and other soft tissue masses 7. On ultrasound, both a pseudoaneurysm and true aneurysm appears as a hypoechoic (dark) cystic mass adjacent to an artery. Application of color Doppler typically reveals pulsatile flow and, in some cases, swirling of the red and blue signals (i.e. the “yin-yang sign”) 8. To best visualize flow in a pseudoaneurysm with color Doppler, the operator can tilt the probe slightly, thereby increasing the Doppler shift.

Patients with pseudoaneurysms or true aneurysms of the foot risk hemorrhage, distal thromboembolism, and compression neuropathy, and should be urgently referred to a podiatrist or vascular surgeon for operative repair 1, 9.

## Conclusion

Emergency physicians should consider lower extremity pseudoaneurysms in patients presenting with soft tissue masses after trauma, even if minor. To our knowledge, this is the first reported case of a pseudoaneurysm of the dorsalis pedis artery diagnosed on point-of-care ultrasound performed by an emergency physician. The identification of a pseudoaneurysm of the dorsalis pedis artery should prompt urgent referral to a surgical specialist to avoid hemorrhage, distal thromboembolism, or neurovascular compromise.

## Disclosures

The authors have no conflicts of interest to declare.

## Supplementary Material

Video S1 Point-of-care ultrasound with Color Doppler demonstrates turbid pulsatile flow (arrow) between the dorsalis pedis artery (A) and adjacent hypoechoic mass (asterisk), consistent with a pseudoaneurysm of the dorsalis pedis artery with thrombus. The dorsalis pedis vein (V) is also visualized.
